# Moth responses to sympathetic hedgerow management in temperate farmland

**DOI:** 10.1016/j.agee.2018.10.008

**Published:** 2019-02-01

**Authors:** Jérémy S.P. Froidevaux, Moth Broyles, Gareth Jones

**Affiliations:** University of Bristol, School of Biological Sciences, Life Sciences Building, 24 Tyndall Avenue, BS8 1TQ, Bristol, United Kingdom

**Keywords:** Agri-environment schemes, Bat, Landscape connectivity, Lepidoptera, Linear features, Woodland

## Abstract

Hedgerows provide valuable habitats and corridors for many species in farmland, yet a lack of appropriate management may threaten their benefits to biodiversity. Although agri-environment scheme (AES) prescriptions on hedgerow management have the potential to reverse the detrimental effect of over-trimming on wildlife, their effectiveness has rarely been addressed. The aims of the study were to (i) assess moth responses to trimming regimes; and (ii) investigate the influence of the surrounding landscape on moth assemblages. We specifically tested the effectiveness of the trimming regime recommended by the targeted AES that was implemented on farms near greater horseshoe bat (*Rhinolophus ferrumequinum*) colonies since it represented the most sympathetic hedgerow management option among English AES options. We sampled adult micro- and macro-moths along 64 hedgerows located within 20 English farms using light traps, and classified moths into two guilds reflecting their larval food preferences, namely grass/herb- and shrub/tree-feeders. Our results suggest that reducing trimming has a positive impact on macro-moth species richness as well as on shrub/tree-feeder abundance and species richness. It also benefited four moth species that are significantly declining in Britain. Furthermore, while the proportion of woodland at a large spatial scale (3.0 km radius around the sampling sites) was positively associated with the abundance of macro-moths and grass/herb-feeders, woodland connectivity had a positive effect on the species richness of grass/herb- and shrub/tree-feeders at large and medium (1.5 km radius) scales, respectively. Both the abundance and species richness of macro-moths and the abundance of shrub/tree-feeders were negatively affected by the presence of arable fields adjacent to hedgerows. Overall, these findings reveal the wider biodiversity benefits of targeted AESs focusing on habitat improvement for *R. ferrumequinum*, and the importance of woodland in the wider landscape. We therefore strongly recommend implementing a multi-scale management approach (i.e. from field to landscape) through the use of adequate AES prescriptions to conserve moths in agricultural landscapes.

## Highlights

• Reducing hedgerow trimming frequency benefits macro-moths and shrub/tree-feeders (all moths species combined).
• Abundance of four nationally-declining moth species was higher on hedgerows left untrimmed for at least three years.
• Increasing woodland amount and connectivity may enhance moth abundance and diversity.
• Arable fields adjacent to hedgerows may have a negative impact on hedgerow-dependent moths.
• Moth conservation in farmland requires a multi-scale management approach.

## 1 Introduction

The expansion and intensification of agricultural land use over the last 60 years has resulted in a dramatic change in agricultural landscapes (Robinson and Sutherland, 2002; Tscharntke et al., 2005). In Europe, hedgerow removal has been one of the first direct consequences of the changes in farming practices as it allowed farmers to increase field size to operate larger machinery (Baudry et al., 2000). The loss of field boundaries has largely contributed to the severe population declines of farmland species (Newton, 2004; Burns et al., 2016) and has affected ecosystem services such as pest control and pollination (Bianchi et al., 2006; Dainese et al., 2017). In addition to reducing both landscape heterogeneity (Benton et al., 2003) and connectivity (Burel, 1996), hedgerow removal has also reduced breeding habitat and food resources at the local scale for many species (e.g. Newton, 2004). Although some countries have since implemented legislation to halt this widespread loss (e.g. the United Kingdom with its Hedgerows Regulation 1997 legislation), new subtler issues regarding the management of hedgerows have emerged. Among these, unsympathetic management (e.g. over-trimming) conducted at most hedgerows is currently deteriorating their structure, thus threatening their existence and benefits to biodiversity (Hinsley and Bellamy, 2000; Carey et al., 2008).

Sensitive management of hedgerows has been mainly promoted in Europe through agri-environment schemes (AESs). This financial incentive program aims to reverse negative biodiversity trends in farmland by encouraging farmers to adopt environmentally sustainable farming practices. In England, the Environmental and Countryside Stewardships operating successively from 2005 onwards include different options to promote sympathetic management of hedgerows (Natural England, 2013a,b, 2016), especially with regards to trimming regime and period. In 2013, over 200,000 km of hedgerows were managed under these agreements (Natural England, 2013a). However, although many studies have investigated how to improve the biodiversity benefits of hedgerows and have provided recommendations to maximize the success of these schemes (see review by Graham et al., 2018), there is still a lack of evidence whether the options on hedgerow management implemented are effective in achieving their intended outcomes. According to the few studies that have empirically tested their effectiveness, it would seem that these prescriptions have had mixed success (Fuentes-Montemayor et al., 2011a, b; Baker et al., 2012; Staley et al., 2016). Additional evidence is therefore urgently needed to improve the design of future AES options and counteract the impacts of unsympathetic management on biodiversity.

Dramatic declines in moth (Lepidoptera) populations in Europe (Conrad et al., 2006; Groenendijk and Ellis, 2011) — partly due to agricultural intensification (Merckx et al., 2012a; Fox, 2013) — have led in recent years to increased interest in their conservation (New, 2004; Warren and Bourn, 2011). Moths represent one of the most species-rich insect groups. They constitute the main prey items of many insectivorous bird and bat species (Vaughan, 1997; Wilson et al., 1999) and provide important ecosystem services including pollination (Devoto et al., 2011; Knop et al., 2017). Sensitive to agricultural intensification (Pocock and Jennings, 2008; Merckx et al., 2012a) and climate change (Fox et al., 2014), moths are indicators of environmental change in terrestrial ecosystems (New, 2004; Rakosy and Schmitt, 2011; Merckx et al., 2013).

Hedgerows are a key habitat for many moth species as they provide food and shelter for both larval and adult stages (Merckx and Macdonald, 2015). They may also act as dispersal pathways (Coulthard et al., 2016) and corridors between woodland patches for moths (Slade et al., 2013). Although Fuentes-Montemayor et al. (2011a) revealed that the sympathetic hedgerow management prescribed by Scottish AESs (trimming restricted to once every three years at specific time of the year) was ineffective in promoting adult moth populations, Staley et al. (2016) found, in contrast, that some aspects of similar schemes in England enhance both abundance and diversity of Lepidoptera larvae and pupae. Similarly, Facey et al. (2014) emphasized that hedgerows trimmed once every two or three years harboured a greater abundance of concealed moth larvae such as leaf miners and case bearers than ones trimmed annually. Although these contrasting results underline complex responses of moth communities to hedgerow management, it remains unclear why adult moths do not benefit from sympathetic management while their larvae do. As demonstrated by Merckx et al. (2012a), grouping moths according to their mobility (sedentary vs. mobile species) and their larval feeding guilds (grass/herb-feeders vs. shrub/tree-feeders) may be crucial in examining the effectiveness of such schemes. Less mobile species are more likely to be affected by local management (Merckx et al., 2010a) compared with more mobile ones that are mainly influenced by landscape features (Slade et al., 2013). Furthermore, shrub/tree-feeders might be more directly affected by hedgerow management than grass/herb-feeders as reducing trimming regimes would increase shelter opportunities, egg-laying sites, and food resource availability for this guild (Merckx et al., 2012a; Staley et al., 2016).

Here, we aimed to assess whether hedgerow management implemented in AESs is effective in enhancing adult moth populations in temperate farmland and determine the influence of landscape-scale factors to that extent. We were particularly interested in investigating the effects of trimming regimes prescribed by the targeted Higher Level Stewardship (HLS; contract of 10 years established between 2005 and 2014) implemented in England. This HLS prescription (HB11/12: “management of hedgerows of very high environmental value”; Natural England, 2013b) specified to trim hedgerows no more than once every three calendar years, to avoid trimming all hedgerows in the same year, and to trim hedgerows between 31 December and 28 February only (Appendix A). It represented the most sympathetic hedgerow management option within the English Environmental Stewardship and has partly been retained in the new Countryside Stewardship (Natural England, 2016). Thus, our objectives were to (i) investigate the effects of trimming regime (i.e. time since last trimming) and landscape characteristics on the abundance and species richness of micro-moths (low-mobility species), macro-moths (high-mobility species), grass/herb-feeders and shrub/tree-feeders; and (ii) examine changes in moth community composition in response to trimming regime. Because HLS prescriptions were implemented in targeted high-priority areas where the most threatened farmland species occur (Natural England, 2013b), we conducted our study on farms where sympathetic hedgerow management was mainly carried out to enhance greater horseshoe bat (*Rhinolophus ferrumequinum*) populations. Hence, this study design also allowed us to assess whether a targeted AES that mainly focused on a single threatened species may have wider biodiversity benefits as suggested by other studies (MacDonald et al., 2012a, b; Wilkinson et al., 2012; Helden et al., 2015).

## 2 Material and methods

### 2.1 Study design

The study was carried out between June and August 2016 on 20 pastoral and mixed farms located in south-west England. Within each farm, we selected hedgerows that were under Higher Level Stewardship (HLS) prescription (hereafter referred to as HLS hedgerows) and matched them with one or several conventionally-managed ones (i.e. trimmed once every one or two calendar years; hereafter referred to as CM hedgerows). We defined a hedgerow as a woody linear feature (i.e. dominated by shrub and tree species) that forms part of a management unit (Baudry et al., 2000). We applied several criteria to appropriately match the hedgerows, specifically considering the hedgerow length (minimum of 100 m), the terrain slope, and the distance to the nearest broadleaf woodland patch. This resulted in the selection of 30 HLS and 34 CM hedgerows. Hedgerows within farms were separated at least 200 m from each other.

### 2.2 Moth sampling and identification

We captured nocturnal moths using a portable heath-type actinic light trap (6 W 12 V actinic bulb; see Froidevaux et al. (2018) for characteristics of the light trap) installed 1 m out from the edge of the midpoint of the hedgerow. The light trap was placed upon a 1 × 1 m white sheet with egg-trays to increase capture rate. Considering the relatively low attraction radius (<30 m) of the light trap (Truxa and Fiedler, 2012; Merckx and Slade, 2014) and the location of the sampling sites (≥50 m from hedgerow nodes), potential biases arising from the capture of moths present along other hedgerows or woodland edges were minimised. Hedgerows located within the same farm were simultaneously sampled once — from 30 min before sunset to 4 h after sunrise — when weather conditions were optimal (i.e. no rain, temperature at sunset > 10 °C, wind speed <30 km/h). Temperature at night was also recorded every 15 min with a data logger (RC-5; accuracy: 0.5 °C; Elitech, London, UK). Although we acknowledge that several sampling nights would have been required to adequately assess the local moth assemblage, we used this single-visit design to maximize the number of sites sampled. This strategy has been adopted in some other research studies on moths when comparing different habitat management methods (e.g. Fuentes-Montemayor et al., 2011a). At the end of the survey, moths resting on the sheet were caught using a sweep net and put in the trap. We dropped a cotton wool ball soaked in ethyl acetate into the trap and sealed it for >10 h to euthanise the individuals captured. Moths were then stored within a −18 °C freezer until identification. We used information from Sterling and Parsons (2012) and Waring and Townsend (2009) to (i) identify moths to the lowest taxonomic level; (ii) assign them to either micro- or macro-moth; and (iii) when possible, classify them into two guilds according to their larval foodplant preferences, namely grass/herb- and shrub/tree-feeders. Although we recognise that the separation of micro- and macro-moth is arbitrary and does not reflect phylogenetic affiliations, this classification may nevertheless relate to species mobility as macro-moths display higher mobility than non-migrant micro-moth species (Nieminen et al., 1999). The distinction between grass/herb- and shrub/tree-feeders (all moth species combined) at the larval stage was made to reflect affinity for woody habitats.

### 2.3 Hedgerow and landscape characteristics

We classified hedgerows according to their trimming regime into three categories: (i) hedgerows that were trimmed the winter prior to sampling (CM hedgerow: N = 24; HLS hedgerow: N = 4); (ii) hedgerows trimmed two winters prior to sampling (CM hedgerow: N = 10; HLS hedgerow: N = 7); and (iii) hedgerows not trimmed for at least three consecutive winters (HLS hedgerow: N = 19). To assess hedgerow compositional and structural variations among the three categories, we conducted field surveys along a 21 m transect parallel to the hedgerow with its centre situated at the sampling site (i.e. midpoint of the hedgerow). The transect was long enough to obtain a reliable representation of the hedgerow characteristics. We divided the transect in 14 equal length sections and identified woody plant species present within each of them. Hedgerow height (including the bank) was measured using a laser distance meter (Tacklife LDM03; accuracy: 2 mm; Shenzen Temie Technology Co., Shenzen, China) at each section boundary (i.e. 15 measures) and width of the hedgerow canopy was calculated at the midpoint of the hedgerow. We characterized the land type adjacent to the hedgerows in two categories, namely (i) grassland, when both fields consisted to either pastures or meadows; and (i) arable land, when at least one field was used for crop production.

We used ArcGIS Desktop v10 (ESRI, Redlands, California, USA) to construct three buffers (0.5, 1.5, and 3.0 km radii) around the sampling sites. These three spatial scales were selected to cover species-specific foraging distances travelled by non-migratory moths in agricultural landscapes (Merckx et al., 2009a, a; Slade et al., 2013). To assess the effects of intensive agriculture on moths (Merckx et al., 2012a), we extracted within each buffer the amount of arable land (Land Cover Map 2015; Rowland et al., 2017). Similarly, as the amount and configuration of broadleaf woodland patches may shape moth communities in farmland-dominated landscapes (Fuentes-Montemayor et al., 2012; Slade et al., 2013), we (i) quantified the proportion of woodland within each buffer; (ii) derived a connectivity index from Fragstats 4.2 (McGarigal et al., 2002) using the mean Euclidean nearest neighbour distance (ENN) between woodland patches at different spatial scales; and (iii) calculated the distance between each sampling site and the nearest respective woodland patch.

### 2.4 Statistical analyses

Statistical analyses were conducted in R v3.4.1 (R Development Core Team, 2017). To test for differences in hedgerow composition and structural variation between the three hedgerow categories, we fitted a series of linear mixed-effects models (LMMs; “lme4″ package; Bates et al., 2015) with trimming regime (time since last trimming) as a fixed effect and farm as a random effect. Response variables (i.e. mean hedgerow height, standard deviation of height, width, and woody plant species richness) were, when necessary, log-transformed beforehand to meet model assumptions. LMMs were followed by Tukey's post hoc multiple comparison tests (“multcomp” package; Hothorn et al., 2008) to examine pairwise differences between treatments.

To disentangle the effects of trimming regime (time since last trimming) and landscape characteristics on the abundance and species richness of (i) micro- and macro-moths; and (ii) grass/herb- and shrub/tree-feeders, we performed a series of generalized linear mixed-effects models (GLMMs; “lme4″ package”). We either used Poisson or negative binomial distribution to handle overdispersion. The interactions of trimming regime and land type surrounding the hedgerows (grassland vs. arable land) alongside landscape attributes were considered as fixed effects while farm was included as a random effect to take into account similarities in farm management between hedgerows present within the same farm. As weather conditions and seasonality may affect nightly catches of moths (Jonason et al., 2014), we also included temperature at night and Julian day as covariates. All continuous variables were beforehand standardized (i.e. rescaled to the same unit) to enable comparisons of effect sizes. Prior to inclusion of landscape attributes into the models, we assessed independently the relationship between each landscape feature with each of the response variables using GLMMs, and selected only the most relevant spatial scale (i.e. the scale in which the variable had the largest effect size). We also undertook a Principal Component Analysis (PCA) with mean, minimum, and maximum temperatures at night and considered the first PCA axis as the temperature variable in our models given that it accounted for 95% of the variance. After inclusion of all variables within the most complex models, we evaluated multicollinearity with the variance inflation factor (VIF; all variables had VIF values <3 indicating no strong correlation between the explanatory variables) and model validation was undertaken using the “DHARMa” package (Hartig, 2017). We generated all possible model combinations using the *dredge* function (“MuMIn” package; Bartoń, 2016) and then restricted our model set such that trimming regime was included in all models. As we were primarily interested to compare hedgerows with different trimming regime, this method allowed us to fix the variable of interest during the model selection process (Grueber et al., 2011). To identify the most parsimonious models, we used the Akaike information criterion corrected for small sample size (*AICc*). When models were found to be equivalent (Δ*AICc* <2), we selected the one having the fewest number of predictors (Burnham and Anderson, 2002). Tukey's post hoc multiple comparison tests were used to investigate pairwise differences between the three trimming categories. Finally, as some HLS hedgerows were not trimmed for 3 to ≥10 years prior to sampling, our study design also allowed us to assess the long-terms effects of non-trimming on moths. We therefore conducted a series of generalized additive mixed models (GAMMs; “mgcv” package; Wood, 2017) using the same model structure as the GLMMs but considering time since last trimming as a continuous (fixed) variable. Variables present in the most parsimonious GLMMs were included as covariates in the GAMMs.

To analyse the influence of trimming regime on moth community composition, we fitted a multivariate generalized linear model (GLM) with the *manyglm* function (“mvabund” package; Wang et al., 2012) using a negative binomial distribution to account for mean-variance relationships and overdispersion of the data. The *manyglm* function fits a specified GLM to each species with a common set of predictors and applies a resampling method to make community-level inferences. This model-based approach of analysing multivariate abundance data has proved to outperform traditional distance-based methods (Warton et al., 2012). Trimming regime was included as the main explanatory variable into the model while temperature at night and Julian day were treated as covariates. Because *manyglm* cannot handle random terms, we also included spatial coordinates of each sampling site as covariates to indicate the proximity of hedgerows belonging to the same farm and therefore consider that some variations in the data may be explained by the farm environment itself. Nevertheless, due to the strong correlation between longitude and latitude coordinates (Spearman’s rank correlation, *r*_s_ = 0.93, *d.f.* = 62, *P* <  0.001), we only kept the latter within the model. Singleton species (N = 65) were removed as they have little effect on the community composition, on statistical outcomes and moreover allows us to reduce the number of species to test, hence minimising issues regarding multiple testing. This resulted in 140 species in the moth database. Model assumptions were checked using diagnostic plots (Warton et al., 2012). We examined the significance of the explanatory variables with the log-likelihood ratio test (LRT) statistic; *P*-values were estimated using the PIT-trap resampling method with 999 resampling iterations (Warton et al., 2017). Pairwise comparisons between hedgerow categories were obtained using post hoc tests. Because we found that species composition significantly differed across hedgerow categories, we then investigated species-specific responses to trimming regime to identify which species differ the most. We decided to assess significance of comparisons using 95% confidence intervals (CIs) of model coefficients rather than *P*-values (Nakagawa and Cuthill, 2007). Reporting adjusted *P*-values for multiple testing would be meaningless (Moran, 2003) due to the high number of species tested (i.e. 140 species). Thus, comparisons were considered as statistically significant when the 95% CIs did not overlap zero. Finally, we extracted from Fox et al. (2013) the British population trends of the moth species between 1968 and 2007 that were significantly influenced by trimming regime.

## 3 Results

### 3.1 Hedgerow structure and composition

**Fig. 1:**
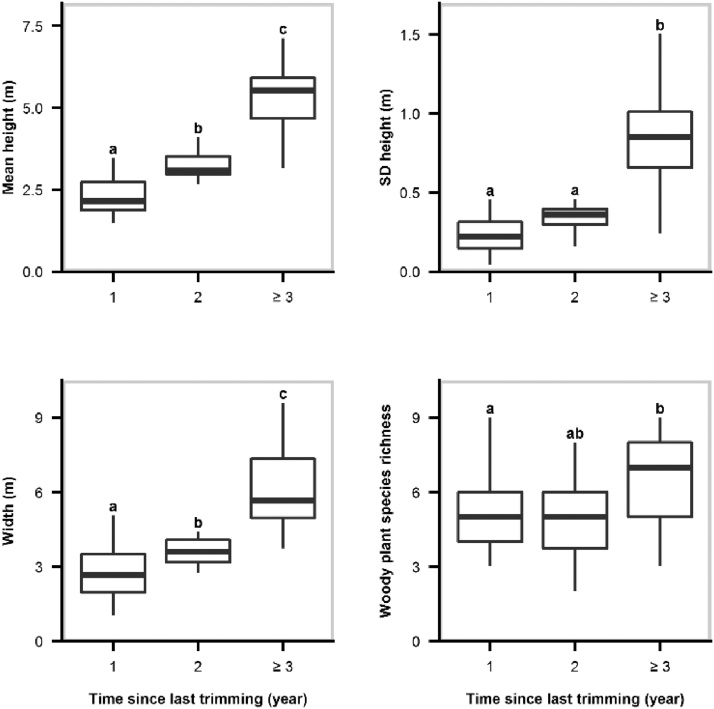
Boxplot of the hedgerow structural and compositional characteristics in relation to trimming regime categories (time since last trimming). Statistically significant differences between treatments are displayed with superscripts.

Hedgerows that were not trimmed for at least three consecutive winters prior to sampling were significantly taller, wider, and more structurally diverse than their recently trimmed counterparts (*P* <  0.001 for all multiple comparisons; Fig. 1). Likewise, our results suggest that hedgerows trimmed the winter prior to sampling harboured significantly fewer woody plant species than those that were trimmed at least three years prior to sampling (*P* =  0.022). Blackthorn (*Prunus spinosa*), hawthorn (*Crataegus* spp.), and hazel (*Corylus avellana*) were the main species found across hedgerow categories. Tree species such as ash (*Fraxinus excelsior*), oak (*Quercus* spp.), and hazel were less frequent in the hedgerows that had just been trimmed.

### 3.2 Effects of trimming regime, land type, and landscape characteristics on moth abundance and species richness

We captured 3234 individual moths belonging to 205 taxa and 25 families (Table A1). Of these, 92% were identified to species level, 7% to species complex, and 1% to genus level. The most abundant 10 taxa (*Oligia fasciuncula*, *Hepialus lupulinus*, *Plutella xylostella* (a migratory species), *Diarsia rubi*, *Scoparia pyralella*-*ambigualis*, *Crambus lathoniellus*, *Agrotis exclamationis*, *Chrysoteuchia culmella*, *Epirrhoe alternate*, and *Ochropleura plecta*) comprised 49% of all collected moths. Macro-moths largely dominated the assemblage with 2213 individuals collected (68%) corresponding to 148 taxa and 10 families. We classified 63 taxa comprising 350 individuals as shrub/tree-feeders and 94 taxa (2206 individuals) as grass/herb-feeders (Table A1).

**Fig. 2:**
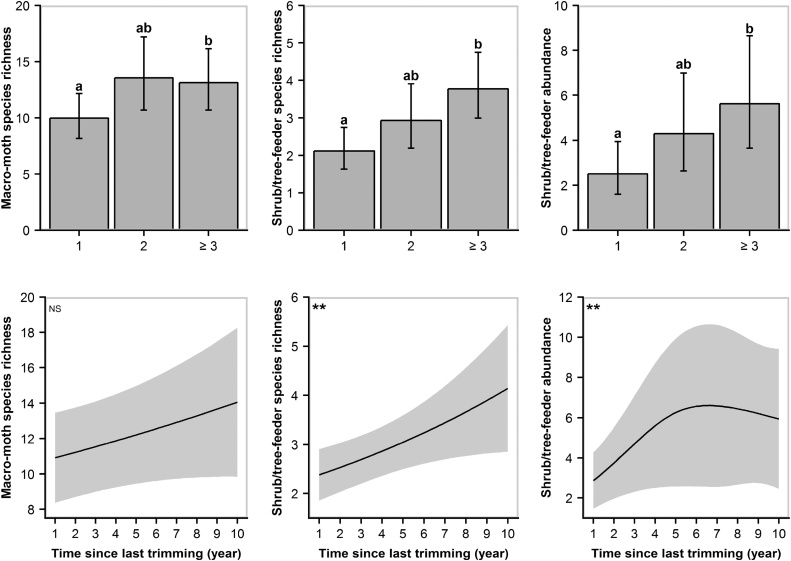
GLMMs (top panel) and GAMMs (bottom panel) predictions and associated 95% confidence intervals on the effects of trimming regime (time since last trimming) on macro−moth species richness and shrub/tree−feeder (micro− and macro−moth species combined) species richness and abundance. Trimming regime was considered as a categorical variable in GLMMs and as a continuous one in GAMMs. Top panel: statistically significant differences between treatments are displayed with superscripts. Bottom panel: statistical significance is displayed at the top left corner of the graph (NS: *P* ≥ 0.10; **P < 0.01).

**Fig. 3:**
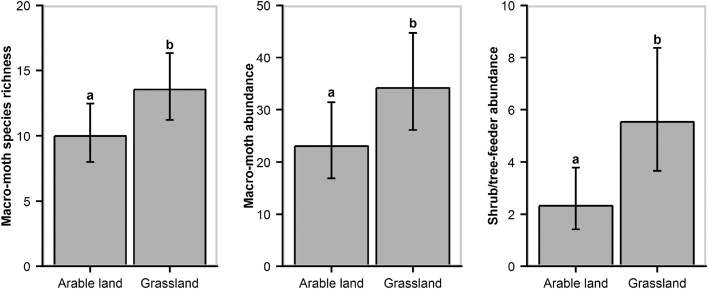
Predicted means and associated 95% confidence intervals of macro−moth species richness, macro−moth abundance, and shrub/tree−feeder (micro− and macro−moths combined) abundance in relation to land type (grassland vs. arable land) adjacent to hedgerows. Predictions arise from the most parsimonious GLMMs. Statistically significant differences between treatments are displayed with superscripts.

**Fig. 4:**
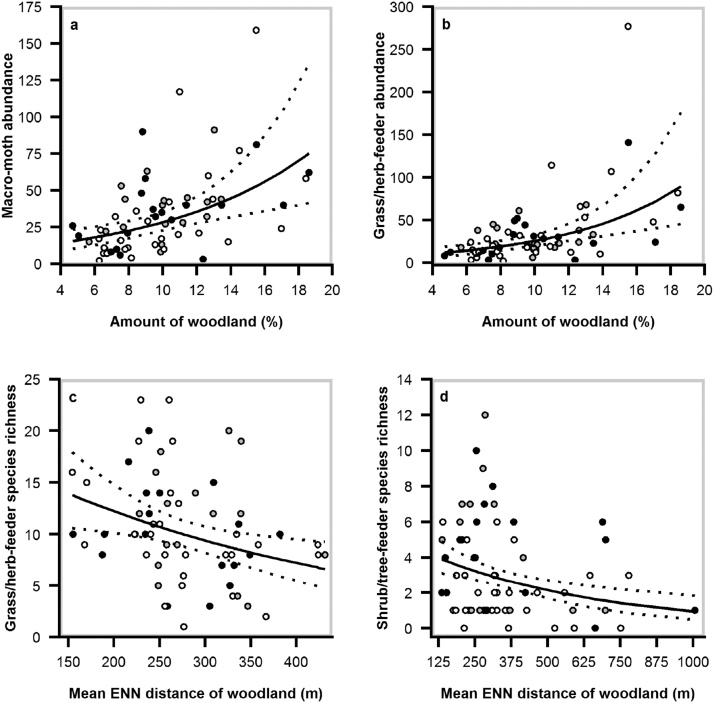
Predicted effects of (i) amount of woodland on (a) macro-moth abundance and (b) grass/herb-feeder abundance; and (ii) mean ENN distance of woodland patches (connectivity index) on (c) grass/herb-feeder species richness and (d) shrub/tree-feeder species richness. The spatial scales of each landscape attribute are indicated in Table 1. Model predictions from GLMMs are represented by the black solid lines with 95% confidence interval indicated by the dotted lines. Open circles: hedgerow trimmed the winter prior to sampling (category 1); filled grey circles: hedgerow trimmed two winters prior to sampling (category 2); black filled circles: hedgerow not trimmed for at least three consecutive winter (category ≥3).

Macro-moths and shrub/tree-feeder moths (micro- and macro-moth species combined) responded positively toward sympathetic hedgerow management (Fig. 2). Macro-moth species richness increased by 32% on hedgerows not trimmed for at least three consecutive winters compared with those trimmed annually. The same pattern was observed for shrub/tree-feeder species richness and abundance with an increase of 79% and 123%, respectively (Table 1). Nevertheless, when looking at the long-term effects of non-trimming, only shrub/tree-feeders abundance and species-richness were positively related to time since last trimming (Fig. 2; Table A2). Our models also revealed that hedgerows surrounded by grassland rather than arable land enhanced macro-moth species richness and abundance by 35% and 48%, respectively. Similarly, the abundance of shrub/tree-feeder moths was predicted to be more than double along hedgerows surrounded by grassland compared with arable land (Fig. 3). The amount of woodland at the largest spatial scale (3.0 km radius) positively influenced the abundance of both macro-moths and grass/herb-feeders (micro- and macro-moth species combined) while woodland connectivity had a significant positive effect on species richness of grass/herb- and shrub/tree-feeders at medium (1.5 km radius) and large scales, respectively (Table 1; Fig. 4). Micro-moth abundance and species richness were only strongly affected by temperature at night (Table 1).

**Table 1:** Results of the most parsimonious GLMMs built to assess the effects of landscape characteristics, land type surrounding the hedgerows and trimming regime on moth abundance and species richness. Results of the Tukey's post hoc multiple comparison tests are displayed for the variable time since last trimming (TSLT). Marginal *R*^2^ (variance explained by the fixed effects only; Nakagawa and Schielzeth, 2013) of each model is given as well as the standardized estimates (effect size), standard errors (SE), test statistics (*Z* value), and *P*−values of each variable. Large (3.0 km radius) and medium (1.5 km radius) spatial scales of the landscape attributes are shown with the superscripts a and b, respectively. The full description of the best models is presented in Table A3.

Response variable	Explanatory variable	Estimate (± SE)	*Z* value	*P*
Micro-moth abundance†	TSLT: 2 vs. 1	0.04 (± 0.29)	0.15	NS
marginal *R*^2^ = 0.44	TSLT: ≥3 vs. 1	−0.30 (± 0.23)	−1.27	NS
	TSLT: ≥3 vs. 2	−0.34 (± 0.29)	−1.18	NS
	Temperature	0.55 (± 0.17)	3.30	***
Micro-moth species richness‡	TSLT: 2 vs. 1	0.00 (± 0.15)	0.03	NS
marginal *R*^2^ = 0.39	TSLT: ≥3 vs. 1	−0.29 (± 0.15)	−1.91	NS
	TSLT: ≥3 vs. 2	−0.29 (± 0.17)	−1.69	NS
	Temperature	0.37 (± 0.09)	4.29	***
Macro-moth abundance†	TSLT: 2 vs. 1	0.34 (± 0.21)	1.59	NS
marginal *R*^2^ = 0.63	TSLT: ≥3 vs. 1	0.23 (± 0.18)	1.23	NS
	TSLT: ≥3 vs. 2	−0.12 (± 0.22)	−0.52	NS
	Grassland vs. arable land	0.39 (± 0.19)	2.09	*
	% woodland^a^	0.37 (± 0.11)	3.51	***
Macro-moth species richness†	TSLT: 2 vs. 1	0.31 (± 0.14)	2.27	·
marginal *R*^2^ = 0.37	TSLT: ≥3 vs. 1	0.28 (± 0.11)	2.41	*
	TSLT: ≥3 vs. 2	−0.03 (± 0.13)	−0.25	NS
	Grassland vs. arable land	0.30 (± 0.13)	2.27	*
	Temperature	0.18 (± 0.08)	2.25	*
Grass/herb-feeder abundance†	TSLT: 2 vs. 1	0.13 (± 0.22)	0.61	NS
marginal *R*^2^ = 0.50	TSLT: ≥3 vs. 1	−0.02 (± 0.19)	−0.09	NS
	TSLT: ≥3 vs. 2	−0.15 (± 0.23)	−0.67	NS
	% woodland^a^	0.47 (± 0.12)	3.93	***
Grass/herb-feeder species richness†	TSLT: 2 vs. 1	0.19 (± 0.13)	1.45	NS
marginal *R*^2^ = 0.39	TSLT: ≥3 vs. 1	0.04 (± 0.12)	0.32	NS
	TSLT: ≥3 vs. 2	−0.15 (± 0.14)	−1.11	NS
	Mean ENN distance of woodland^a^	−0.16 (± 0.06)	−2.71	**
	Temperature	0.24 (± 0.06)	3.80	***
Shrub/tree-feeder abundance†	TSLT: 2 vs. 1	0.54 (± 0.24)	2.19	·
marginal *R*^2^ = 0.30	TSLT: ≥3 vs. 1	0.80 (± 0.20)	3.99	***
	TSLT: ≥3 vs. 2	0.27 (± 0.22)	1.22	NS
	Grassland vs. arable land	0.87 (± 0.24)	3.55	***
Shrub/tree-feeder species richness‡	TSLT: 2 vs. 1	0.32 (± 0.20)	1.64	NS
marginal *R*^2^ = 0.54	TSLT: ≥3 vs. 1	0.58 (± 0.17)	3.35	**
	TSLT: ≥3 vs. 2	0.25 (± 0.18)	1.39	NS
	Julian day	0.28 (± 0.08)	3.29	**
	Mean ENN distance of woodland^b^	−0.30 (± 0.09)	−3.42	***
	Temperature	0.46 (± 0.08)	5.51	***

NS: *P* ≥ 0.10; ·*P* <  0.10; * *P* < 0.05; ** *P* <  0.01; *** *P* <  0.001.

† GLMMs with negative binomial distribution.

‡ GLMMs with Poisson distribution.

### 3.3 Effects of trimming regime on moth community composition

After accounting for spatial aggregation of hedgerows (*P* <  0.001), Julian day (*P* <  0.001), and temperature at night (*P* <  0.001), multivariate GLM analysis indicated that moth community composition significantly differs across trimming regime categories (*P* =  0.012; Table A4). Only 73 species (including singleton ones), representing 36% of total number of species, were shared between the three types of hedgerow (Figure A1). When investigating species-specific responses to trimming regime, 16 taxa were found to be strongly affected (Table 2) including seven that are significantly declining in Britain (Fox et al., 2013). Of these, the abundance of four species (*Cosmorhoe ocellata*, *Eulithis pyraliata*, *Lomographa temerata*, and *Peribatodes rhomboidaria*) was enhanced by hedgerows that remained untrimmed for at least two consecutive winters, two species (*Acronicta rumicis* and *Ennomos fuscantaria*) were significantly more abundant on hedgerows not trimmed for at least three winters compared with hedgerows trimmed two winters prior to sampling, and one species (*Mythimna pallens*) was more abundant along the most recently trimmed hedgerows (Table 2). Nevertheless, these results should be interpreted with caution as spurious relationships (false positives) may occur due to the high number of species tested (140).

**Table 2:** Results of the multivariate GLM built to investigate individual species responses to trimming regime. Only species that significantly differ across treatments are shown. Pairwise comparisons were considered as statistical significant if the 95% confidence intervals of the modelled estimate did not overlap zero. Population trends of moths in Britain between 1969 and 2007 were extracted from Fox et al. (2013).

Taxa	Treatment	Estimate (± SE)	Confidence interval	Population trend (1968-2007)
*Abraxas grossulariata*	2 vs. 1	2.38 (± 1.12)	(0.18, 4.58)	Slightly declining (−21%)
*Colostygia pectinataria*	2 vs. 1	2.65 (± 1.31)	(0.08, 5.22)	Significantly increasing (+230%)
*Cosmorhoe ocellata*	2 vs. 1	2.65 (± 1.22)	(0.26, 5.04)	Significantly declining (−22%)
*Epirrhoe alternata*	2 vs. 1	1.80 (± 0.50)	(0.82, 2.78)	Slightly increasing (+19%)
*Eulithis pyraliata*	2 vs. 1	1.64 (± 0.77)	(0.13, 3.15)	Significantly declining (−54%)
*Idaea seriata*	2 vs. 1	1.47 (± 0.61)	(0.27, 2.67)	Significantly increasing (+155%)
*Noctua pronuba*	2 vs. 1	1.35 (± 0.45)	(0.47, 2.23)	Significantly increasing (+186%)
*Peribatodes rhomboidaria*	2 vs. 1	1.26 (± 0.60)	(0.08, 2.44)	Significantly declining (−48%)
*Udea olivalis*	2 vs. 1	1.38 (± 0.62)	(0.16, 2.60)	NA
*Abraxas grossulariata*	≥3 vs. 1	2.97 (± 1.11)	(0.79, 5.15)	Slightly declining (−21%)
*Cabera pusaria*	≥3 vs. 1	2.94 (± 1.02)	(0.94, 4.94)	Significantly increasing (+86%)
*Lomographa temerata*	≥3 vs. 1	1.85 (± 0.90)	(0.09, 3.61)	Significantly declining (−48%)
*Mythimna pallens*	≥3 vs. 1	−3.65 (± 1.64)	(-6.86, -0.44)	Significantly declining (−59%)
*Peribatodes rhomboidaria*	≥3 vs. 1	1.60 (± 0.55)	(0.52, 2.68)	Significantly declining (−48%)
*Scoparia pyralella-ambigualis*	≥3 vs. 1	−1.99 (± 0.57)	(-3.11, -0.87)	NA
*Acronicta rumicis*	≥3 vs. 2	4.89 (± 1.35)	(2.24, 7.54)	Significantly declining (−75%)
*Cabera pusaria*	≥3 vs. 2	2.63 (± 1.15)	(0.38, 4.88)	Significantly increasing (+86%)
*Celypha lacunana*	≥3 vs. 2	−2.36 (± 0.95)	(-4.22, -0.50)	NA
*Colostygia pectinataria*	≥3 vs. 2	−3.41 (± 1.40)	(-6.15, -0.67)	Significantly increasing (+230%)
*Ennomos fuscantaria*	≥3 vs. 2	1.79 (± 0.71)	(0.40, 3.18)	Significantly declining (−98%)
*Epirrhoe alternata*	≥3 vs. 2	−0.88 (± 0.44)	(-1.74, -0.02)	Slightly increasing (+19%)
*Scoparia pyralella-ambigualis*	≥3 vs. 2	−2.05 (± 0.80)	(-3.62, -0.48)	NA

## 4 Discussion

Overall, our study shows that sympathetic hedgerow management may benefit macro-moths, shrub/tree-feeder moths, and some moth species that significantly declined in Britain over the last decades. These results highlight the wider positive impact of targeted AESs that primarily focus on enhancing populations of the greater horseshoe bat (*Rhinolophus ferrumequinum*). Our results also deepen our understanding of the main landscape drivers determining moth abundance and diversity along hedgerows and emphasize the detrimental effect of intensive farming at local scale. Many moth species have suffered severe population declines during the last decades (Conrad et al., 2006; Groenendijk and Ellis, 2011; Fox et al., 2013) and our findings contribute to the implementation of effective conservation measures that favour moths in farmland.

### 4.1 Effects of trimming regime

Although hedgerows are widely assumed to be beneficial to butterflies and moths in farmland (Maudsley, 2000; Boutin et al., 2011), their value and importance seem to be determined by their management (Merckx and Berwaerts, 2010; Graham et al., 2018). When investigating the responses of adult macro-moths to trimming regime, our results indicated a positive relationship between macro-moth species richness and time since last trimming. Hedgerows left untrimmed for at least three winters were taller, wider, and structurally more diverse and may therefore provide better shelters to moths by improving microclimate conditions (Maudsley, 2000; Merckx et al., 2008). These findings are in line with Merckx et al., 2009b, Merckx et al., 2010a, Merckx et al., 2012a who highlighted the shelter effect of hedgerow trees for macro-moths in exposed agricultural landscapes. Untrimmed hedgerows may also enhance food provisions to adult moths that depend on nectar resources. Staley et al. (2012) emphasized that floral resource availability was considerably greater in hedgerows left untrimmed for three years compared with annually trimmed ones.

Regarding micro-moths, there were no significant differences in species richness and abundance between trimming regime categories, thus corroborating the findings of Fuentes-Montemayor et al. (2011a). Merckx et al., 2009a, Merckx et al., 2010a demonstrated that less mobile moth species were more prone to be affected by local management, yet we found that low mobility species such as micro-moths were only influenced by temperature at night while high mobility species such as macro-moths were affected by hedgerow management. This might certainly highlight some limitations in using these two species groups (i.e. micro- and macro-moths) to reflect differences in species mobility as mobility can also be determined by other factors than wingspan such as wing shape, habitat affinity, and migratory behaviour (Slade et al., 2013). It is important to point out that the results were similar if micro- and macro-moths are categorised into larval feeding groups, and if migratory species are removed (Table A5).

The classification of moths into two feeding guilds according to their larval foodplant preferences allowed us to highlight the significant positive effect of sympathetic trimming regime on the abundance and species richness of shrub/tree-feeders. Given that moth larvae and pupae occurring on woody hedgerow plants also benefit from sympathetic trimming regimes (Staley et al., 2016), our results demonstrate the crucial importance of untrimmed hedgerows in providing adequate habitats and resources to shrub/tree-feeders during their full life cycle. Hedgerows left untrimmed for at least three winters are more likely to fulfil species requirements of shrub/tree-feeders as they may harbour more host plants than over-trimmed ones and provide more egg-laying sites and larval food resources. Besides increasing hedgerow structural diversity, our results indicated that the implementation of sympathetic trimming regime also enhanced woody plant species richness within hedgerows.

When assessing species-specific responses to trimming regime, we found that hedgerows left untrimmed for at least three years enhanced the abundance of four species that significantly declined during the last decades. Of these, two are listed as 'Priority under section 41 of the NERC Act', namely the knot grass (*Acronicta rumicis*) and the dusky thorn (*Ennomos fuscantaria*). These species have declined by 75% and 98%, respectively, between 1968 and 2007 in Britain (Fox et al., 2013). The implementation of sympathetic hedgerow management proves therefore to be crucial for the conservation of these species in farmland.

### 4.2 Influence of the surrounding environment

Landscape variables related to the proportion of broadleaf woodland at medium (1.5 km radius) and large (3.0 km radius) scales were key drivers of the abundance of macro-moths and grass/herb-feeders and the diversity of both shrub/tree- and grass/herb-feeders guilds. Broadleaf woodlands constitute important habitat for moths as many species depend on them at different stages of their life cycle (Waring and Townsend, 2009; Sterling and Parsons, 2012; Merckx, 2015). They provide food resources at both larval and adult stages (Summerville and Crist, 2004), offer essential shelter (Merckx et al., 2012b), and may act as population sources in agricultural landscapes (Ricketts et al., 2001). Given the strong affinity of shrub/tree-feeders to woodland (Summerville and Crist, 2004), it is not surprising to find a significant effect of woodland connectivity on their species richness. This finding somewhat concurs with that of Slade et al. (2013), who found that forest specialists were dependent on woodland connectivity to move through the agricultural matrix. Moreover, the amount and connectivity of woodlands might also be beneficial to grass/herb-feeders as they may enhance shelter and plant host diversity in the landscape (Merckx, 2015). The positive relationship found in Britain between species richness of herbaceous forest plants and woodland patch size and connectivity (Petit et al., 2004) supports this hypothesis.

The abundance and diversity of macro-moths were negatively impacted by the presence of arable land in the fields adjacent to hedgerows. When assessing the effects of grassland restoration on moths, Alison et al. (2017) found similar outcomes with fewer moth individuals recorded on arable fields compared with fields restored to species-rich grassland and semi-natural calcareous grassland. Most of the grassland fields (57%) in our study consisted of pastures grazed by either sheep or cattle. Grazing may have a detrimental impact on moth communities by increasing habitat disturbance and reducing food larval resources (Littlewood, 2008; van Klink et al., 2015) but our results suggest that arable land may have an even stronger negative impacts on macro-moth species which might be related to biotic homogenisation caused by land-use changes (Ekroos et al., 2010). This was also observed by Fuentes-Montemayor et al. (2012), who caught fewer species and moth individuals in woodland patches surrounded by arable land than in those surrounded by pastures. Although we did not find any evidence of a landscape-scale effect, other studies have underlined the broader impact of land-use intensification on moths. For instance, Fox et al. (2014) pointed out that the decline of widespread moth species in southern England was partly associated with the increased of arable land cover. Similarly, Merckx et al. (2012a) showed that nationally declining macro-moth species in Britain were most strongly impacted by the amount of arable land at medium spatial scale (0.8 km). Lastly, shrub/tree-feeders were in our study also found to be less abundant along hedgerows surrounded by arable fields. While this finding seems to be surprising as this guild primarily depends on woody habitats, it may reflect the potential wider negative impact of insecticide applications in croplands on non-targeted insects in adjacent habitats since hedgerows prove to be very effective in intercepting spray drift (Lazzaro et al., 2008).

### 4.3 Implications for moth and bat conservation

This study provides strong evidence of the value of targeted AESs in improving habitat conditions of non-target species as suggested by other studies (MacDonald et al., 2012a, b; Wilkinson et al., 2012; Helden et al., 2015). While the conservation of moths in farmland may require specific tailored management actions (but see Merckx et al., 2010b), our findings indicate that moths may also benefit from sympathetic hedgerow management targeting *R. ferrumequinum*, a bat species of major conservation concerns. Importantly, the fact that moths constitute a major component of the diet of this bat (Vaughan, 1997) might explain the success of AES prescriptions on hedgerow management in enhancing *R. ferrumequinum* in farmland (Froidevaux et al., unpublished data), thus highlighting the crucial importance of improving field-scale management practices to increase prey availability for bats. Since populations of many bat species have suffered drastic declines in Western Europe during the second half of the 20^th^ century partly due to reduction in insect populations caused by agricultural intensification (Stebbings, 1988), conservation measures that effectively enhance moth populations in farmland are likely to benefit bat species that prey on them.

In accordance with other studies (Facey et al., 2014; Staley et al., 2016), our results point out the need for reducing hedgerow trimming frequency to favour moths that use hedgerows. Nevertheless, trimming once every two winters as partly prescribed by the Countryside Stewardship in England (BE3 option: "Management of hedgerows"; Natural England, 2016) may not be enough to promote adult moth abundance and diversity. Our findings largely support the long-term benefits of non-trimming on shrub/tree-feeder moths. We therefore strongly recommend avoiding trimming hedgerows for at least three years to maximize the biodiversity benefits of sympathetic hedgerow management. Furthermore, although hedgerows sampled in our study were trimmed during winter only, the most common practice of trimming hedgerows in early autumn proves to be detrimental to moths (Staley et al., 2016). Hence, when trimming is needed, it is vital to (i) trim hedgerows during winter time only; and (ii) avoid trimming all hedgerows during the same year to maintain their benefits to biodiversity within the farm across years.

It is now well recognized that a landscape-scale management approach is required to promote moth populations in agricultural landscapes (Fuentes-Montemayor et al., 2011a; Merckx and Macdonald, 2015), yet there is still not a consensus among studies regarding the spatial scales at which moths may benefit the most from this approach (Ricketts et al., 2001; Fuentes-Montemayor et al., 2011a, 2012; Merckx et al., 2012a). Due to the high variability in species mobility across moth species (Slade et al., 2013), it is important to implement a multi-scale approach that takes into account the range of dispersion of the species of interest (Gonthier et al., 2014; Merckx et al., 2018). We recommend maintaining and increasing the amount and connectivity of broadleaf woodland within the agricultural matrix at medium (1.5 km radius) and large (3.0 km radius) spatial scales while restoring arable fields into semi-natural grassland at the farm scale (Alison et al., 2017). Many AES options in England and elsewhere may provide financial incentives to these purposes (Fuentes-Montemayor et al., 2011a; Natural England, 2016). We finally recommend future studies to consider the combined effect of hedgerow management and landscape characteristics in delivering biodiversity benefits and ecosystem services in agricultural landscapes.

## Funding

BBSRCPP Grant no: 1700589
