# Supplementary material for: Moth responses to sympathetic hedgerow management in temperate farmland
Source: Agric Ecosyst Environ. 2019 Feb 1;270-271:55–64. doi: 10.1016/j.agee.2018.10.008 (PMC6472680; doi:10.1016/j.agee.2018.10.008)
Supplement: Supplementary file 1 [file mmc1.docx]

Supplementary Data

Moth responses to sympathetic hedgerow management in temperate farmland

Jérémy S. P. Froidevaux, Moth Broyles, and Gareth Jones

**Appendix A1.** Description of the targeted agri-environment prescription HB11/12 “maintenance of hedges of very high environmental value”.

1. *General description of the management required*

This option manages hedgerows that support target species of farmland birds, insects or mammals. It also maintains hedgerows which make a significant contribution to the local landscape character and/or are historically important boundaries.

1. *Indicators of success*

- Each year, there should be some uncut hedgerows on the holding.
- By year 5, hedges should be at least 3 m in height overall (measured from the ground on the lower side); with at least 1.5m height of shrubby growth and 2 m width of shrubby growth above the top of the bank.
- By year 5 there should be 3 to 10 hedgerow trees for every 100 m of hedge at irregular spacings.
- Greater horseshoe bats fly along the hedges during the summer months.
- Branches and shrubby growth is allowed to overhang the adjacent field to create an umbrella-shaped canopy for parts of the hedge.

1. *Management prescriptions*

The following rules apply across the whole area being managed under this option.

- Allow hedges to reach and then maintain the dimensions described in the *indicators of success* above by moving the flail a little further out with each trim.
- Trim hedges between 31 December and 28 February only.
- Trim hedges no more than one year in three. Trimming of hedges should be rotated to avoid cutting all hedges in the same year.
- Where there are insufficient hedgerow trees to meet the *indicators of success* described above, plant or tag hedgerow trees. If planting new trees, use native and locally common species.
- Retain all mature growth of ivy on trees.
- Retain all standing deadwood unless it presents a genuine safety hazard.

1. *Payment*

- £54 per 100 m (both sides)
- £27 per 100 m (one side)

**Table A1.** Summary table of the number of moths collected along the three categories of hedgerows.

| **Taxa** | **Larval**  **feeding guild** | **Time since last trimming (year)** | | | **Total** |
| --- | --- | --- | --- | --- | --- |
|  |  | **1**  (N = 28) | **2**  (N = 17) | **≥3**  (N = 19) |  |
| *Macro-moth* |  | *892* | *635* | *686* | *2213* |
| *Abraxas grossulariata* | shrub/tree-feeders | 1 | 8 | 11 | 20 |
| *Abrostola tripartita* | grass/herb-feeders | 2 | 1 | 0 | 3 |
| *Acronicta aceris* | shrub/tree-feeders | 1 | 0 | 0 | 1 |
| *Acronicta leporina* | shrub/tree-feeders | 1 | 0 | 0 | 1 |
| *Acronicta megacephala* | shrub/tree-feeders | 0 | 1 | 0 | 1 |
| *Acronicta rumicis* |  | 0 | 1 | 10 | 11 |
| *Agrotis clavis* | grass/herb-feeders | 1 | 0 | 0 | 1 |
| *Agrotis exclamationis* | grass/herb-feeders | 58 | 27 | 37 | 122 |
| *Agrotis ipsilon* | grass/herb-feeders | 1 | 1 | 0 | 2 |
| *Alcis repandata* | shrub/tree-feeders | 3 | 3 | 3 | 9 |
| *Apamea crenata* | grass/herb-feeders | 2 | 0 | 0 | 2 |
| *Apamea lithoxylaea* | grass/herb-feeders | 1 | 1 | 1 | 3 |
| *Apamea monoglypha* | grass/herb-feeders | 18 | 25 | 15 | 58 |
| *Apamea sordens* | grass/herb-feeders | 2 | 0 | 0 | 2 |
| *Autographa gamma* | grass/herb-feeders | 1 | 2 | 3 | 6 |
| *Autographa jota* |  | 3 | 2 | 0 | 5 |
| *Axylia putris* | grass/herb-feeders | 5 | 3 | 12 | 20 |
| *Biston betularia* |  | 2 | 0 | 0 | 2 |
| *Cabera exanthemata* | shrub/tree-feeders | 5 | 2 | 6 | 13 |
| *Cabera pusaria* | shrub/tree-feeders | 2 | 2 | 5 | 9 |
| *Calliteara pudibunda* | shrub/tree-feeders | 2 | 0 | 4 | 6 |
| *Campaea margaritata* | shrub/tree-feeders | 3 | 0 | 0 | 3 |
| *Camptogramma bilineata* | grass/herb-feeders | 4 | 1 | 1 | 6 |
| *Caradrina morpheus* |  | 0 | 0 | 1 | 1 |
| *Catarhoe cuculata* | grass/herb-feeders | 0 | 1 | 0 | 1 |
| *Catarhoe rubidata* | grass/herb-feeders | 0 | 2 | 0 | 2 |
| *Cerapteryx graminis* | grass/herb-feeders | 0 | 3 | 1 | 4 |
| *Charanyca trigrammica* | grass/herb-feeders | 12 | 1 | 15 | 28 |
| *Chloroclysta truncata* |  | 6 | 4 | 3 | 13 |
| *Chloroclystis v-ata* |  | 1 | 0 | 1 | 2 |
| *Chortodes pygmina* | grass/herb-feeders | 0 | 0 | 1 | 1 |
| *Cidaria fulvata* | grass/herb-feeders | 0 | 3 | 3 | 6 |
| *Cilix glaucata* | shrub/tree-feeders | 0 | 1 | 0 | 1 |
| *Cleorodes lichenaria* |  | 2 | 0 | 1 | 3 |
| *Colostygia pectinataria* | grass/herb-feeders | 15 | 20 | 4 | 39 |
| *Cosmorhoe ocellata* | grass/herb-feeders | 1 | 8 | 5 | 14 |
| *Craniophora ligustri* | shrub/tree-feeders | 0 | 0 | 3 | 3 |
| *Crocallis elinguaria* | shrub/tree-feeders | 2 | 4 | 5 | 11 |
| *Cyclophora punctaria* | shrub/tree-feeders | 0 | 1 | 1 | 2 |
| *Deilephila elpenor* | grass/herb-feeders | 0 | 5 | 0 | 5 |
| *Diachrysia chrysitis* | grass/herb-feeders | 4 | 2 | 0 | 6 |
| *Diarsia brunnea* |  | 0 | 0 | 4 | 4 |
| *Diarsia mendica* |  | 0 | 0 | 7 | 7 |
| *Diarsia rubi* |  | 54 | 78 | 41 | 173 |
| *Discoloxia blomeri* | shrub/tree-feeders | 1 | 0 | 0 | 1 |
| *Drepana falcataria* | shrub/tree-feeders | 0 | 1 | 1 | 2 |
| *Ecliptopera silaceata* | grass/herb-feeders | 1 | 0 | 0 | 1 |
| *Eilema griseola* |  | 10 | 8 | 9 | 27 |
| *Eilema lurideola* |  | 5 | 1 | 11 | 17 |
| *Eilema sororcula* |  | 0 | 0 | 1 | 1 |
| *Electrophaes corylata* | shrub/tree-feeders | 1 | 0 | 0 | 1 |
| *Ennomos alniaria* | shrub/tree-feeders | 0 | 0 | 1 | 1 |
| *Ennomos fuscantaria* | shrub/tree-feeders | 0 | 3 | 6 | 9 |
| *Epirrhoe alternata* | grass/herb-feeders | 9 | 52 | 17 | 78 |
| *Eulithis pyraliata* | grass/herb-feeders | 4 | 7 | 6 | 17 |
| *Euphyia unangulata* |  | 0 | 0 | 1 | 1 |
| *Eupithecia linariata* | grass/herb-feeders | 3 | 0 | 1 | 4 |
| *Eupithecia tenuiata* | shrub/tree-feeders | 0 | 2 | 0 | 2 |
| *Eupithecia tripunctaria* |  | 0 | 0 | 1 | 1 |
| *Euplagia quadripunctaria* |  | 0 | 2 | 0 | 2 |
| *Euplexia lucipara* |  | 0 | 1 | 1 | 2 |
| *Euthrix potatoria* | grass/herb-feeders | 2 | 1 | 3 | 6 |
| *Gortyna flavago* | grass/herb-feeders | 0 | 1 | 0 | 1 |
| *Habrosyne pyritoides* | shrub/tree-feeders | 0 | 2 | 0 | 2 |
| *Hadena bicruris* | grass/herb-feeders | 0 | 0 | 1 | 1 |
| *Hepialus lupulinus* | grass/herb-feeders | 177 | 7 | 74 | 258 |
| *Hepialus sylvina* | grass/herb-feeders | 0 | 3 | 1 | 4 |
| *Herminia grisealis* | shrub/tree-feeders | 0 | 0 | 1 | 1 |
| *Hoplodrina alsines* | grass/herb-feeders | 0 | 6 | 7 | 13 |
| *Hoplodrina blanda* | grass/herb-feeders | 4 | 6 | 2 | 12 |
| *Horisme tersata* | grass/herb-feeders | 7 | 0 | 1 | 8 |
| *Horisme vitalbata* | grass/herb-feeders | 1 | 0 | 0 | 1 |
| *Hydraecia micacea* | grass/herb-feeders | 0 | 3 | 3 | 6 |
| *Hydrelia flammeolaria* | shrub/tree-feeders | 0 | 0 | 1 | 1 |
| *Hydriomena furcata* | shrub/tree-feeders | 12 | 3 | 6 | 21 |
| *Hypena proboscidalis* | grass/herb-feeders | 7 | 8 | 9 | 24 |
| *Idaea aversata* | grass/herb-feeders | 3 | 8 | 5 | 16 |
| *Idaea biselata* | grass/herb-feeders | 4 | 1 | 1 | 6 |
| *Idaea dimidiata* | grass/herb-feeders | 2 | 0 | 0 | 2 |
| *Idaea seriata* |  | 9 | 9 | 7 | 25 |
| *Idaea* spp. |  | 0 | 1 | 0 | 1 |
| *Idaea subsericeata* | grass/herb-feeders | 1 | 0 | 0 | 1 |
| *Lacanobia oleracea* |  | 0 | 0 | 1 | 1 |
| *Laothoe populi* | shrub/tree-feeders | 0 | 0 | 1 | 1 |
| *Laspeyria flexula* |  | 1 | 1 | 0 | 2 |
| *Ligdia adustata* | shrub/tree-feeders | 1 | 2 | 1 | 4 |
| *Lomaspilis marginata* | shrub/tree-feeders | 3 | 0 | 5 | 8 |
| *Lomographa temerata* | shrub/tree-feeders | 6 | 3 | 10 | 19 |
| *Lycophotia porphyrea* | shrub/tree-feeders | 0 | 1 | 1 | 2 |
| *Lymantria monacha* | shrub/tree-feeders | 0 | 0 | 2 | 2 |
| *Macaria alternata* | shrub/tree-feeders | 1 | 3 | 3 | 7 |
| *Macaria liturata* | shrub/tree-feeders | 0 | 0 | 1 | 1 |
| *Malacosoma neustria* | shrub/tree-feeders | 1 | 5 | 2 | 8 |
| *Mamestra brassicae* |  | 1 | 0 | 0 | 1 |
| *Mesapamea secalis-didyma* | grass/herb-feeders | 11 | 20 | 9 | 40 |
| *Mesoligia furuncula* | grass/herb-feeders | 2 | 3 | 0 | 5 |
| *Mormo maura* |  | 0 | 1 | 0 | 1 |
| *Mythimna comma* | grass/herb-feeders | 3 | 0 | 4 | 7 |
| *Mythimna conigera* | grass/herb-feeders | 0 | 1 | 0 | 1 |
| *Mythimna ferrago* | grass/herb-feeders | 1 | 1 | 2 | 4 |
| *Mythimna impura* | grass/herb-feeders | 14 | 12 | 5 | 31 |
| *Mythimna pallens* | grass/herb-feeders | 8 | 20 | 1 | 29 |
| *Mythimna straminea* | grass/herb-feeders | 0 | 1 | 0 | 1 |
| *Mythimna turca* | grass/herb-feeders | 0 | 0 | 1 | 1 |
| *Noctua comes* |  | 1 | 2 | 0 | 3 |
| *Noctua interjecta caliginosa* |  | 0 | 3 | 1 | 4 |
| *Noctua janthe* |  | 1 | 22 | 3 | 26 |
| *Noctua pronuba* | grass/herb-feeders | 9 | 31 | 14 | 54 |
| *Notodonta dromedarius* | shrub/tree-feeders | 0 | 2 | 0 | 2 |
| *Nudaria mundana* |  | 1 | 0 | 0 | 1 |
| *Ochropleura plecta* | grass/herb-feeders | 13 | 41 | 24 | 78 |
| *Oligia fasciuncula* | grass/herb-feeders | 190 | 4 | 73 | 267 |
| *Oligia strigilis-versicolor-latruncula* | grass/herb-feeders | 27 | 4 | 11 | 42 |
| *Opisthograptis luteolata* | shrub/tree-feeders | 0 | 15 | 11 | 26 |
| *Orthonama obstipata* | grass/herb-feeders | 1 | 0 | 0 | 1 |
| *Ourapteryx sambucaria* | shrub/tree-feeders | 0 | 1 | 0 | 1 |
| *Peribatodes rhomboidaria* | shrub/tree-feeders | 7 | 17 | 21 | 45 |
| *Perizoma affinitata* | grass/herb-feeders | 2 | 2 | 2 | 6 |
| *Perizoma albulata* | grass/herb-feeders | 12 | 0 | 10 | 22 |
| *Perizoma alchemillata* | grass/herb-feeders | 6 | 1 | 2 | 9 |
| *Perizoma flavofasciata* | grass/herb-feeders | 0 | 1 | 0 | 1 |
| *Petrophora chlorosata* |  | 1 | 0 | 0 | 1 |
| *Phragmatobia fuliginosa* |  | 1 | 0 | 4 | 5 |
| *Plagodis dolabraria* | shrub/tree-feeders | 0 | 0 | 1 | 1 |
| *Plusia festucae* | grass/herb-feeders | 1 | 1 | 0 | 2 |
| *Protodeltote pygarga* | grass/herb-feeders | 0 | 0 | 1 | 1 |
| *Pseudoips prasinana britannica* | shrub/tree-feeders | 0 | 0 | 1 | 1 |
| *Rivula sericealis* | grass/herb-feeders | 10 | 8 | 8 | 26 |
| *Rusina ferruginea* | grass/herb-feeders | 4 | 1 | 4 | 9 |
| *Scopula imitaria* |  | 1 | 0 | 1 | 2 |
| *Scotopteryx chenopodiata* | grass/herb-feeders | 1 | 0 | 0 | 1 |
| *Selenia dentaria* | shrub/tree-feeders | 3 | 5 | 6 | 14 |
| *Sphinx ligustri* | shrub/tree-feeders | 1 | 1 | 0 | 2 |
| *Spilosoma lubricipeda* | grass/herb-feeders | 24 | 0 | 14 | 38 |
| *Spilosoma luteum* |  | 9 | 5 | 16 | 30 |
| *Stauropus fagi* | shrub/tree-feeders | 0 | 0 | 1 | 1 |
| *Thumatha senex* |  | 1 | 0 | 0 | 1 |
| *Timandra comae* | grass/herb-feeders | 5 | 6 | 1 | 12 |
| *Triphosa dubitata* | shrub/tree-feeders | 1 | 1 | 0 | 2 |
| *Watsonalla binaria* | shrub/tree-feeders | 0 | 2 | 1 | 3 |
| *Xanthorhoe designata* | grass/herb-feeders | 1 | 0 | 1 | 2 |
| *Xanthorhoe fluctuata* | grass/herb-feeders | 1 | 1 | 3 | 5 |
| *Xanthorhoe montanata* | grass/herb-feeders | 10 | 5 | 5 | 20 |
| *Xanthorhoe spadicearia* | grass/herb-feeders | 2 | 4 | 3 | 9 |
| *Xestia c-nigrum* | grass/herb-feeders | 10 | 8 | 7 | 25 |
| *Xestia sexstrigata* |  | 0 | 5 | 0 | 5 |
| *Xestia triangulum* |  | 1 | 3 | 0 | 4 |
| *Xestia xanthographa* | grass/herb-feeders | 0 | 5 | 4 | 9 |
| *Zanclognatha tarsipennalis* | shrub/tree-feeders | 3 | 5 | 1 | 9 |
| *Micro-moth* |  | *571* | *216* | *234* | *1021* |
| *Acleris laterana* | shrub/tree-feeders | 0 | 2 | 0 | 2 |
| *Agapeta hamana* | grass/herb-feeders | 8 | 2 | 7 | 17 |
| *Agriphila straminella* | grass/herb-feeders | 4 | 14 | 3 | 21 |
| *Agriphila tristella* | grass/herb-feeders | 14 | 40 | 10 | 64 |
| *Alucita hexadactyla* | shrub/tree-feeders | 0 | 0 | 4 | 4 |
| *Anania coronata* | shrub/tree-feeders | 0 | 1 | 1 | 2 |
| *Anania hortulata* | grass/herb-feeders | 2 | 1 | 2 | 5 |
| *Aphomia sociella* |  | 1 | 0 | 0 | 1 |
| *Archips podana* | shrub/tree-feeders | 1 | 0 | 0 | 1 |
| *Argyresthia spinosella* | shrub/tree-feeders | 1 | 0 | 0 | 1 |
| *Blastobasis lacticolella* |  | 1 | 0 | 0 | 1 |
| *Bryotropha terrella* |  | 1 | 5 | 5 | 11 |
| *Cataclysta lemnata* | grass/herb-feeders | 0 | 0 | 1 | 1 |
| *Celypha lacunana* |  | 11 | 33 | 2 | 46 |
| *Celypha striana* | grass/herb-feeders | 1 | 0 | 0 | 1 |
| *Chrysoteuchia culmella* | grass/herb-feeders | 44 | 20 | 31 | 95 |
| *Cnephasia* spp. | grass/herb-feeders | 4 | 1 | 4 | 9 |
| *Cnephasia stephensiana* | grass/herb-feeders | 1 | 0 | 0 | 1 |
| *Coleophora* spp. |  | 2 | 3 | 4 | 9 |
| *Coleophora trifolii* | grass/herb-feeders | 2 | 0 | 0 | 2 |
| *Crambus lathoniellus* | grass/herb-feeders | 107 | 0 | 29 | 136 |
| *Crambus perlella* | grass/herb-feeders | 9 | 0 | 2 | 11 |
| *Cydia splendana* | shrub/tree-feeders | 4 | 0 | 2 | 6 |
| *Depressaria radiella* | grass/herb-feeders | 0 | 4 | 0 | 4 |
| *Epinotia tenerana* | shrub/tree-feeders | 2 | 0 | 0 | 2 |
| *Eucosma hohenwartiana* | grass/herb-feeders | 2 | 0 | 0 | 2 |
| *Eudonia angustea* |  | 4 | 0 | 0 | 4 |
| *Eudonia mercurella* |  | 6 | 10 | 4 | 20 |
| *Eudonia pallida* |  | 11 | 1 | 0 | 12 |
| *Eupoecilia angustana* | grass/herb-feeders | 2 | 1 | 1 | 4 |
| *Euzophera pinguis* | shrub/tree-feeders | 0 | 1 | 0 | 1 |
| *Gypsonoma dealbana* | shrub/tree-feeders | 0 | 1 | 3 | 4 |
| *Hedya pruniana* | shrub/tree-feeders | 9 | 2 | 12 | 23 |
| *Lozotaenia forsterana* | shrub/tree-feeders | 0 | 0 | 1 | 1 |
| *Lyonetia clerkella* | shrub/tree-feeders | 0 | 1 | 0 | 1 |
| *Mecyna asinalis* | grass/herb-feeders | 0 | 1 | 0 | 1 |
| *Metzneria lappella-metzneriella* | grass/herb-feeders | 1 | 0 | 0 | 1 |
| *Myelois circumvoluta* | grass/herb-feeders | 1 | 2 | 0 | 3 |
| *Nematopogon schwarziellus* |  | 0 | 0 | 1 | 1 |
| *Nomophila noctuella* | grass/herb-feeders | 3 | 9 | 1 | 13 |
| *Notocelia trimaculana* | shrub/tree-feeders | 3 | 0 | 1 | 4 |
| *Notocelia uddmanniana* | shrub/tree-feeders | 1 | 2 | 0 | 3 |
| *Orthotaenia undulana* |  | 1 | 0 | 0 | 1 |
| *Pammene fasciana* | shrub/tree-feeders | 2 | 0 | 0 | 2 |
| *Pandemis cerasana* |  | 4 | 12 | 7 | 23 |
| *Parapoynx stratiotata* | grass/herb-feeders | 1 | 0 | 1 | 2 |
| *Pleuroptya ruralis* |  | 1 | 6 | 2 | 9 |
| *Plutella xylostella* | grass/herb-feeders | 153 | 14 | 70 | 237 |
| *Pseudargyrotoza conwagana* | shrub/tree-feeders | 1 | 0 | 0 | 1 |
| *Ptycholoma lecheana* | shrub/tree-feeders | 0 | 1 | 0 | 1 |
| *Scoparia ambigualis* |  | 5 | 0 | 2 | 7 |
| *Scoparia pyralella-ambigualis* |  | 125 | 8 | 7 | 140 |
| *Scrobipalpa costella* | grass/herb-feeders | 0 | 0 | 1 | 1 |
| *Stigmella aurella* |  | 1 | 0 | 0 | 1 |
| *Tachystola acroxantha* |  | 1 | 0 | 0 | 1 |
| *Udea lutealis* | grass/herb-feeders | 4 | 2 | 4 | 10 |
| *Udea olivalis* | grass/herb-feeders | 5 | 9 | 3 | 17 |
| *Udea prunalis* |  | 3 | 0 | 3 | 6 |
| *Yponomeuta malinellus-cagnagella* | shrub/tree-feeders | 1 | 1 | 0 | 2 |
| *Yponomeuta plumbella* | shrub/tree-feeders | 0 | 6 | 3 | 9 |
| **Grand Total** |  | **1463** | **851** | **920** | **3234** |

**Table A2.** Results of the GAMMs built to investigate the long-term effects of non-trimming on macro-moth species richness, shrub/tree-feeder species richness, and shrub/tree-feeder abundance.

Variables present in the most parsimonious GLMMs (see Tables 1 and A3) were included as covariates in the GAMMs. Standardized estimates (effect size), standard errors (SE), test statistics (*F* value for the smooth terms of GAMMs, *t* value otherwise), and *P*-values are given.

| **Response variable** | **Explanatory variable** | **Estimate (± SE)** | **Test statistic** | ***P*** |
| --- | --- | --- | --- | --- |
| Macro-moth species richness^†^ | Time since last trimming | *s* | 3.18 | NS |
|  | Temperature | 0.16 (± 0.09) | 1.65 | NS |
|  | Grassland vs. arable land | 0.36 (± 0.14) | 2.55 | * |
|  |  |  |  |  |
| Shrub/tree-feeder abundance^†^ | Time since last trimming | *s* | 4.94 | ** |
|  | Grassland vs. arable land | 0.95 (± 0.25) | 3.74 | *** |
|  |  |  |  |  |
| Shrub/tree-feeder species richness^‡^ | Time since last trimming | *s* | 7.67 | ** |
|  | Julian day | 0.32 (± 0.09) | 3.56 | *** |
|  | Woodland connectivity (1.5 km) | 0.32 (± 0.09) | 3.47 | *** |
|  | Temperature | 0.45 (± 0.09) | 4.77 | *** |

*s* represents the smooth term of GAMMs.

**^†^** GAMMs with negative binomial distribution; **^‡^** GAMMs with Poisson distribution.

NS: *P* ≥ 0.10; **^.^** *P* < 0.10; * *P* < 0.05; ** *P* < 0.01; *** *P* < 0.001

**Table A3-a.** Description of the most parsimonious GLMMs (*ΔAICc* <2) built to assess the effects of landscape characteristics, land type surrounding the hedgerows, and trimming regime on micro- and macro-moth abundance and species richness. Models are ranked in ascending order of *AICc* and *AICc* weight (*Wt*), cumulative weight (*Cum. Wt*), and marginal *R*^2^ (variance explained by the fixed effects only; Nakagawa and Schielzeth 2013) values are given for each model. The spatial scale of each landscape attribute is given in brackets. The woodland connectivity index corresponds to the mean Euclidean nearest neighbour distance of woodland patches. We restricted the model set during the model selection process such that all models include time since last trimming, the main factor of interest (Grueber *et al.*, 2011). All units are defined in the core text.

| **Model** | ***K*** | ***AICc*** | ***ΔAICc*** | ***AICc Wt*** | ***R*^2^** |
| --- | --- | --- | --- | --- | --- |
| **Micro moth abundance^†^** |  |  |  |  |  |
| Temperature + Time since last trimming + % woodland (3.0 km) | 7 | 465.43 | 0.00 | 0.12 | 0.58 |
| Julian day + Temperature + Time since last trimming | 7 | 465.80 | 0.36 | 0.10 | 0.56 |
| % arable land (3.0 km) + Temperature + Time since last trimming + % woodland (3.0 km) | 8 | 465.96 | 0.52 | 0.09 | 0.67 |
| Julian day + Temperature + Time since last trimming + % woodland (3.0 km) | 8 | 466.20 | 0.77 | 0.08 | 0.63 |
| % arable land (3.0 km) + Temperature + Time since last trimming | 7 | 466.35 | 0.91 | 0.08 | 0.56 |
| Woodland connectivity (3.0 km) + Temperature + Time since last trimming | 7 | 466.50 | 1.07 | 0.07 | 0.46 |
| % arable land (3.0 km) + Woodland connectivity (3.0 km) + Temperature + Time since last trimming | 8 | 466.53 | 1.10 | 0.07 | 0.60 |
| Temperature + Time since last trimming | *6* | *466.58* | *1.15* | *0.07* | *0.44* |
| Woodland connectivity (3.0 km) + Temperature + Time since last trimming + % woodland (3.0 km) | 8 | 466.81 | 1.38 | 0.06 | 0.58 |
| % arable land (3.0 km) + Distance to the nearest woodland + Temperature + Time since last trimming | 8 | 466.91 | 1.47 | 0.06 | 0.61 |
| Distance to the nearest woodland + Temperature + Time since last trimming | 7 | 467.09 | 1.66 | 0.05 | 0.49 |
| Distance to the nearest woodland + Julian day + Temperature + Time since last trimming | 8 | 467.13 | 1.70 | 0.05 | 0.58 |
| Woodland connectivity (3.0 km) + Julian day + Temperature + Time since last trimming | 8 | 467.24 | 1.81 | 0.05 | 0.56 |
| % arable land (3.0 km) + Woodland connectivity (3.0 km) + Temperature + Time since last trimming + % woodland (3.0 km) | 9 | 467.38 | 1.95 | 0.05 | 0.66 |
| **Micro moth species richness^‡^** |  |  |  |  |  |
| Temperature + Time since last trimming | 5 | 291.12 | 0.00 | 0.50 | 0.39 |
| Woodland connectivity (3.0 km) + Temperature + Time since last trimming | 6 | 292.22 | 1.11 | 0.29 | 0.39 |
| % arable land (1.5 km) + Temperature + Time since last trimming | 6 | 292.82 | 1.70 | 0.21 | 0.40 |
| **Macro moth abundance^†^** |  |  |  |  |  |
| Land type + Time since last trimming + % woodland (3.0 km) | **7** | 560.73 | 0.00 | 0.64 | 0.63 |
| % arable land (3.0 km) + Land type + Time since last trimming + % woodland (3.0 km) | 8 | 561.89 | 1.16 | 0.36 | 0.61 |
| **Macro moth species richness^†^** |  |  |  |  |  |
| Temperature + Land type * Time since last trimming | 9 | 407.68 | 0.00 | 0.42 | 0.38 |
| Land type + Temperature + Time since last trimming | **7** | 408.61 | 0.93 | 0.26 | 0.37 |
| Distance to the nearest woodland + Temperature + Land type * Time since last trimming | 10 | 409.63 | 1.95 | 0.16 | 0.39 |
| Temperature + % woodland (3.0 km) + Land type * Time since last trimming | 10 | 409.64 | 1.96 | 0.16 | 0.41 |

**^†^** GLMMs with negative binomial distribution; **^‡^** GLMMs with Poisson distribution.

**References**

Grueber, C.E., Nakagawa, S., Laws, R.J., Jamieson, I.G., 2011. Multimodel inference in ecology and evolution: challenges and solutions. Journal of Evolutionary Biology 24, 699-711.

Nakagawa, S., Schielzeth, H., 2013. A general and simple method for obtaining *R*^2^ from generalized linear mixed-effects models. Methods in Ecology and Evolution 4, 133-142.

**Table A3-b.** Description of the most parsimonious GLMMs (*ΔAICc* <2) built to assess the effects of landscape characteristics, land type surrounding the hedgerows, and trimming regime on grass/herb- and shrub/tree-feeder moth abundance and species richness. Table description as for Table A3-a.

| **Model** | ***K*** | ***AICc*** | ***ΔAICc*** | ***AICc Wt*** | ***R*^2^** |
| --- | --- | --- | --- | --- | --- |
| **Grass/herb-feeder abundance^†^** |  |  |  |  |  |
| Temperature + Time since last trimming + % woodland (3.0 km) | 7 | 553.70 | 0.00 | 0.24 | 0.62 |
| Julian day + Temperature + Time since last trimming + % woodland (3.0 km) | 8 | 554.17 | 0.47 | 0.19 | 0.72 |
| % arable land (3.0 km) + Temperature + Time since last trimming + % woodland (3.0 km) | 8 | 554.42 | 0.72 | 0.16 | 0.74 |
| Time since last trimming + % woodland (3.0 km) | 6 | 554.50 | 0.79 | 0.16 | 0.50 |
| % arable land (3.0 km) + Time since last trimming + % woodland (3.0 km) | 7 | 554.65 | 0.95 | 0.15 | 0.65 |
| Julian day + Time since last trimming + % woodland (3.0 km) | 7 | 555.22 | 1.52 | 0.11 | 0.59 |
| **Grass/herb-feeder species richness^†^** |  |  |  |  |  |
| Woodland connectivity (3.0 km) + Temperature + Time since last trimming | 7 | 379.91 | 0.00 | 0.56 | 0.39 |
| Woodland connectivity (3.0 km) + Land type + Temperature + Time since last trimming | 8 | 381.76 | 1.85 | 0.22 | 0.37 |
| Woodland connectivity (3.0 km) + Temperature + Time since last trimming + % woodland (3.0 km) | 8 | 381.77 | 1.86 | 0.22 | 0.40 |
| **Shrub/tree-feeder abundance^†^** |  |  |  |  |  |
| Woodland connectivity (1.5 km) + Julian day + Land type + Temperature + Time since last trimming | 9 | 327.68 | 0.00 | 0.20 | 0.57 |
| Land type + Temperature + Time since last trimming + % woodland (1.5 km) | 8 | 328.13 | 0.45 | 0.16 | 0.51 |
| Land type + Temperature + Time since last trimming | 7 | 328.21 | 0.53 | 0.15 | 0.44 |
| Woodland connectivity (1.5 km) + Land type + Temperature + Time since last trimming | 8 | 328.44 | 0.76 | 0.14 | 0.48 |
| Land type + Time since last trimming + % woodland (1.5 km) | 7 | 328.91 | 1.23 | 0.11 | 0.40 |
| Land type + Time since last trimming | 6 | 329.28 | 1.60 | 0.09 | 0.30 |
| Woodland connectivity (1.5 km) + Land type + Temperature + Time since last trimming + % woodland (1.5 km) | 9 | 329.55 | 1.87 | 0.08 | 0.53 |
| Julian day + Land type + Temperature + Time since last trimming | 8 | 329.61 | 1.93 | 0.08 | 0.47 |
| **Shrub/tree-feeder species richness^‡^** |  |  |  |  |  |
| Woodland connectivity (1.5 km) + Julian day + Land type + Temperature + Time since last trimming | 8 | 263.84 | 0.00 | 0.66 | 0.51 |
| Woodland connectivity (1.5 km) + Julian day + Temperature + Time since last trimming | 7 | 265.18 | 1.34 | 0.34 | 0.54 |

**^†^** GLMMs with negative binomial distribution; **^‡^** GLMMs with Poisson distribution.

**Table A4.** Results of the multivariate GLM built to investigate community composition to trimming regime (time since last trimming) while accounting for latitude, Julian day, and temperature.

| **Variable** | ***df*** | **Dev** | ***P*** | **Post hoc** |
| --- | --- | --- | --- | --- |
| Latitude | 1 | 600.9 | *** |  |
| Julian day | 1 | 784.6 | *** |  |
| Temperature | 1 | 390.7 | *** |  |
| Time since last trimming | 2 | 411.2 | * | 1 ≠ 2 ≠ ≥3 |

* *P* < 0.05; ** *P* < 0.01; *** *P* < 0.001

**Table A5.** Results of the most parsimonious GLMMs built to assess the effects of landscape characteristics, land type surrounding the hedgerows and trimming regime on the abundance and species richness of non-migrant (i) grass/herb feeding micro-moths; (ii) shrub/tree feeding micro-moths; (iii) grass/herb feeding macro-moths; and (iv) shrub/tree feeding macro-moths. Results of the Tukey's post hoc multiple comparison tests are displayed for the variable time since last trimming (TSLT). Marginal *R*^2^ (variance explained by the fixed effects only; Nakagawa and Schielzeth 2013) of each model is given as well as the standardized estimates (effect size), standard errors (SE), test statistics (*Z* value), and *P*-values of each variable. Large (3.0 km radius) and medium (1.5 km radius) spatial scales of the landscape attributes are given in brackets.

| **Response variable** | **Explanatory variable** | **Estimate (± SE)** | ***Z* value** | ***P*** |
| --- | --- | --- | --- | --- |
| Abundance of grass/herb feeding micro-moths^†^  marginal *R*^2^ = 0.48 | TSLT: 2 vs. 1 | 0.18 (± 0.26) | 0.68 | NS |
|  | TSLT: ≥3 vs. 1 | -0.18 (± 0.22) | -0.81 | NS |
|  | TSLT: ≥3 vs. 2 | -0.36 (± 0.27) | -1.35 | NS |
|  | Temperature | 0.67 (± 0.17) | 4.04 | *** |
| Abundance of shrub/tree feeding micro-moths^†^  marginal *R*^2^ = 0.27 | TSLT: 2 vs. 1 | 0.30 (± 0.43) | 0.7 | NS |
|  | TSLT: ≥3 vs. 1 | 0.66 (± 0.40) | 1.64 | NS |
|  | TSLT: ≥3 vs. 2 | 0.36 (± 0.44) | 0.83 | NS |
|  | Temperature | 0.45 (± 0.20) | 2.21 | * |
| Abundance of grass/herb feeding macro-moths^†^  marginal *R*^2^ = 0.49 | TSLT: 2 vs. 1 | 0.20 (± 0.23) | 0.86 | NS |
|  | TSLT: ≥3 vs. 1 | 0.07 (± 0.20) | 0.34 | NS |
|  | TSLT: ≥3 vs. 2 | -0.13 (± 0.24) | -0.56 | NS |
|  | % woodland (3.0 km) | 0.49 (± 0.13) | 3.83 | *** |
| Abundance of shrub/tree feeding macro-moths^†^  marginal *R*^2^ = 0.51 | TSLT: 2 vs. 1 | 0.73 (± 0.22) | 3.32 | ** |
|  | TSLT: ≥3 vs. 1 | 1.03 (± 0.19) | 5.32 | *** |
|  | TSLT: ≥3 vs. 2 | 0.29 (± 0.18) | 1.66 | NS |
|  | Land type: grassland vs. arable | 0.75 (± 0.23) | 3.31 | *** |
|  | Temperature | 0.46 (± 0.18) | 2.61 | ** |
|  | % arable (3.0 km) | 0.32 (± 0.16) | 1.97 | * |
| Species richness of grass/herb feeding micro-moths^‡^  marginal *R*^2^ = 0.33 | TSLT: 2 vs. 1 | -0.21 (± 0.20) | -1.04 | NS |
|  | TSLT: ≥3 vs. 1 | -0.37 (± 0.20) | -1.86 | NS |
|  | TSLT: ≥3 vs. 2 | -0.16 (± 0.23) | -0.69 | NS |
|  | Temperature | 0.38 (± 0.10) | 3.65 | *** |
| Species richness of shrub/tree feeding micro-moths^‡^  marginal *R*^2^ = 0.15 | TSLT: 2 vs. 1 | 0.20 (± 0.38) | 0.52 | NS |
|  | TSLT: ≥3 vs. 1 | -0.10 (± 0.39) | -0.26 | NS |
|  | TSLT: ≥3 vs. 2 | -0.30 (± 0.42) | -0.71 | NS |
|  | Temperature | 0.41 (± 0.19) | 2.15 | * |
| Species richness of grass/herb feeding macro-moths^‡^  marginal *R*^2^ = 0.32 | TSLT: 2 vs. 1 | 0.28 (± 0.13) | 2.19 | · |
|  | TSLT: ≥3 vs. 1 | 0.13 (± 0.11) | 1.16 | NS |
|  | TSLT: ≥3 vs. 2 | -0.15 (± 0.13) | -1.16 | NS |
|  | Woodland connectivity (3.0 km) | 0.19 (± 0.06) | 3.06 | ** |
|  | Temperature | 0.20 (± 0.07) | 3.13 | ** |
| Species richness of shrub/tree feeding macro-moths^‡^  marginal *R*^2^ = 0.51 | TSLT: 2 vs. 1 | 0.51 (± 0.22) | 2.35 | * |
|  | TSLT: ≥3 vs. 1 | 0.69 (± 0.20) | 3.39 | ** |
|  | TSLT: ≥3 vs. 2 | 0.18 (± 0.19) | 0.94 | NS |
|  | % woodland (1.5 km) | 0.19 (± 0.08) | 2.42 | * |
|  | Temperature | 0.46 (± 0.10) | 4.47 | *** |
|  | % arable (3.0 km) | 0.27 (± 0.09) | 3.01 | ** |

^†^ GLMMs with negative binomial distribution.

^‡^ GLMMs with Poisson distribution.

NS: *P* ≥ 0.10*;* · *P* < 0.10; * *P* < 0.05; ** *P* < 0.01; *** *P* < 0.001


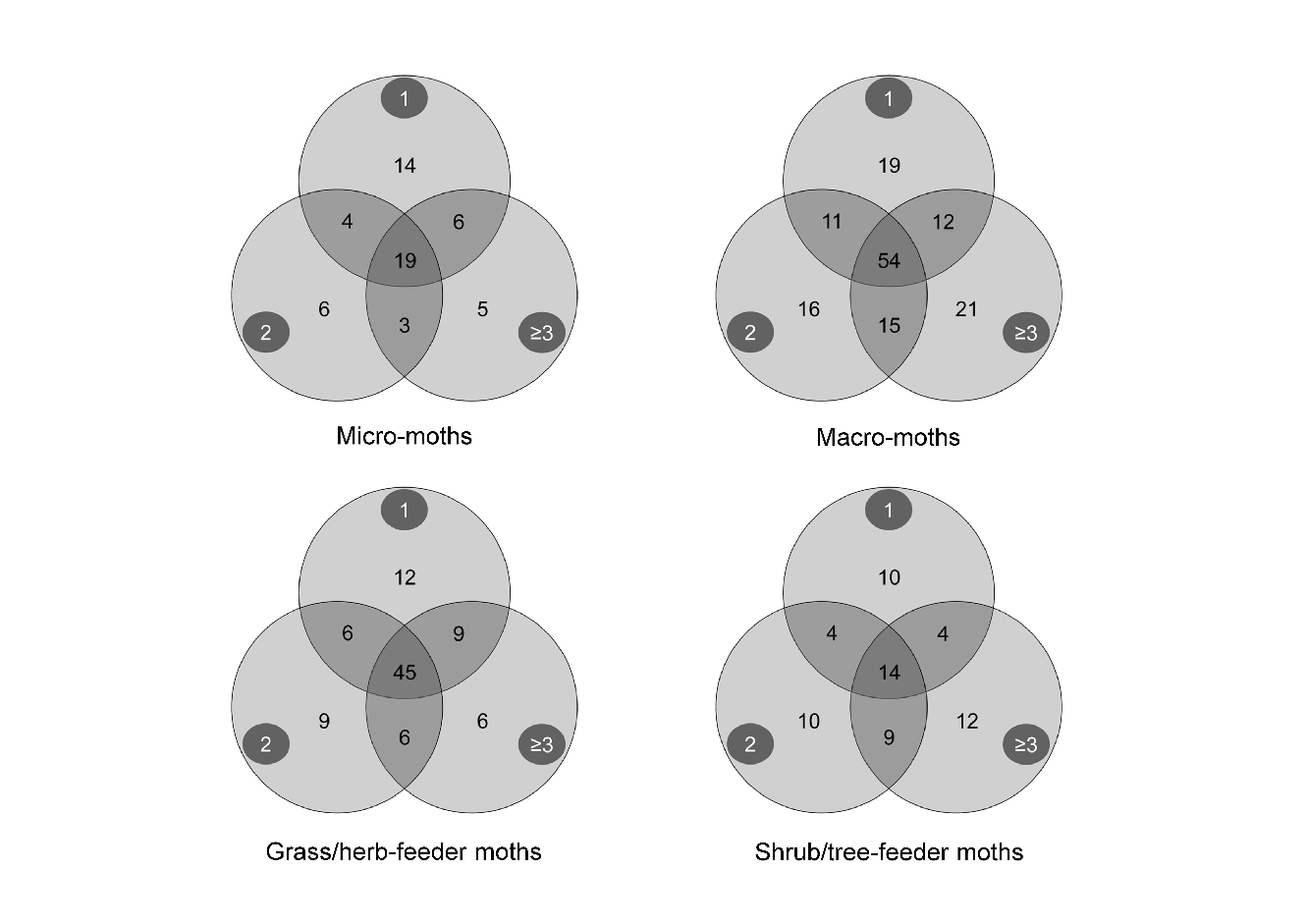


**Figure S1.** Venn diagrams showing the number of moth species of each trimming regime and shared number of species between trimming regimes. 1: hedgerows that were trimmed the winter prior to sampling (N = 28); 2: hedgerows trimmed two winters prior to sampling (N = 17); and ≥3: hedgerows not trimmed for at least three consecutive winters (N = 19).
